# Comment on Althobaiti et al. Potential Association between the Use of Anabolic Steroids and COVID-19 Infection. *Healthcare* 2022, *10*, 196

**DOI:** 10.3390/healthcare10071172

**Published:** 2022-06-23

**Authors:** María-Jimena Muciño-Bermejo

**Affiliations:** 1 International Renal Research Institute of Vicenza, 36100 Vicenza, Italy; srivere.a.jimena@gmail.com or maria.mucinobe@anahuac.mx; 2 American British Cowdray Medical Center, Mexico City 01120, Mexico; 3 North Campus, Anahuac University, Mexico City 52786, Mexico

Recently, Althobaiti, Y. S. and colleagues [1] reported a potential association between the use of anabolic Steroids and COVID-19 infection. First, I would like to congratulate the authors for emphasizing the importance of supplements and over the counter medication on healthy individuals’ homeostasis. Secondly, I would like to mention some technical considerations:

In the introduction section, it would be important to consider that anabolic steroid abuse has reported to be associated with septic complications other than coronavirus diseases, thus giving a clinical antecedent and a biological plausibility to your hypothesis [2].

In addition, to reinforce the plausibility of the causality of anabolic steroids as a worsening factor in COVID-19 disease, according to the Bradford Hill criteria: [3,4,5]

1. Is it possible to prove any biological gradient (i.e., if there were any differences in dose-dependent effect) to stablish a dose-response relationship?
2. As you investigate the temporality of the exposure, it would be interesting to establish a time point in which the history of previous anabolic steroid use no longer has any effect on the COVID-19 clinical course.
3. To remark upon the specificity of the association, you could consider any other supplement use history with a chronological concordance.
4. It would be fruitful to ascertain if there were any relationships between anabolic steroid use and other known risk-factors for COVID-19 disease, such as the weekly amount of time spent in a sport center (i.e., if patients with anabolic steroid use were more likely to spend more time in the sport center/have more social contact) [6].

In addition, regarding the differences reported concerning the clinical symptoms between current users, previous users, and non-users, it would be very interesting to add, if possible, any supplemental information regarding a documented clinical course (i.e., if current anabolic steroids were more likely to develop the renal complications of COVID-19 disease).

Again, I would like to remark on the brilliant approach you have taken to an underestimated public health issue, especially in the context of an increasing “return to normal” in the context of the COVID-19 pandemic.
